# Using the big data approach to clarify the structure of restricted and repetitive behaviors across the most commonly used autism spectrum disorder measures

**DOI:** 10.1186/s13229-021-00419-9

**Published:** 2021-05-27

**Authors:** Mirko Uljarević, Booil Jo, Thomas W. Frazier, Lawrence Scahill, Eric A. Youngstrom, Antonio Y. Hardan

**Affiliations:** 1 Melbourne School of Psychological Sciences, Faculty of Medicine, Dentistry, and Health Sciences, University of Melbourne, Victoria, Australia; 2 Department of Psychiatry and Behavioral Sciences, Stanford University, Stanford, CA USA; 3 Department of Psychology, John Carroll University, University Heights, OH USA; 4 Marcus Autism Center, Emory University School of Medicine, Atlanta, GA USA; 5 Department of Psychology and Neuroscience, University of North Carolina at Chapel Hill, Davie Hall, Chapel Hill, NC USA

**Keywords:** Circumscribed interest, Repetitive motor behavior, Insistence of sameness, Factor analysis, Autism spectrum disorder

## Abstract

**Background:**

Restricted and repetitive behaviors (RRB) in autism spectrum disorder (ASD) encompass several distinct domains. However, commonly used general ASD measures provide broad RRB scores rather than assessing separate RRB domains. The main objective of the current investigation was to conduct a psychometric evaluation of the ability of the Social Responsiveness Scale (SRS-2), the Social Communication Questionnaire (SCQ), the Autism Diagnostic Interview-Revised (ADI-R) and the Autism Diagnostic Observation Schedule (ADOS) to capture different RRB constructs.

**Methods:**

Exploratory Structural Equation Modeling (ESEM) was conducted using individual item-level data from the SRS-2, SCQ, ADI-R and the ADOS. Data were obtained from five existing publicly available databases. For the SRS-2, the final sample consisted of N = 16,761 individuals (*M*_*age*_ = 9.43, *SD* = 3.73; 18.5% female); for the SCQ, of N = 15,840 (*M*_*age*_ = 7.99, *SD* = 4.06; 18.1% female); for the ADI-R, of N = 8985 (*M*_*age*_ = 8.86, *SD* = 4.68; 19.4% female); and for the ADOS, of N = 6314 (*M*_*age*_ = 12.29, *SD* = 6.79; 17.7% female).

**Results:**

The three-factor structure provided the most optimal and interpretable fit to data for all measures (comparative fit index ≥ .983, Tucker Lewis index ≥ .966, root mean square error of approximation ≤ .028). Repetitive-motor behaviors, insistence on sameness and unusual or circumscribed interests factors emerged across all instruments. No acceptable fit was identified for the ADOS.

**Limitations:**

The five datasets used here afforded a large as well as wide distribution of the RRB item scores. However, measures used for establishing convergent and divergent validity were only available for a portion of the sample.

**Conclusions:**

Reported findings offer promise for capturing important RRB domains using general ASD measures and highlight the need for measurement development.

**Supplementary Information:**

The online version contains supplementary material available at 10.1186/s13229-021-00419-9.

## Background

Restricted and repetitive behaviors and interests (RRB) have been recognized as a core symptom domain of autism spectrum disorder (ASD) since the original clinical descriptions by Leo Kanner [1]. RRB can be a major barrier to learning and adaptation, and a source of stress and management challenges for families [2–5]. Despite clinical significance, RRB domain remains poorly understood. Inconsistencies in the organization, division and measurement have had a particularly negative impact on RRB research [4, 6, 7]. Most notably, although recent investigations provide evidence that RRB are not a unitary construct but encompass several distinct domains [8–11], the ability of commonly used diagnostic and quantitative ASD measures to assess these domains separately has not been systematically evaluated. By conflating distinct constructs, overly broad domain scores can cloud the understanding of RRB trajectories, the recognition of associations with other clinical domains and hinder understanding of neurobiological mechanisms. Given the wide use of measures such as the Social Responsiveness Scale-2 (SRS-2 [12]), the Social Communication Questionnaire (SCQ [13]), and the Autism Diagnostic Observation Schedule (ADOS [14]) across research and clinical contexts, and their availability in several large-scale databases that also include neuroimaging and genetic data, rigorous psychometric evaluation of the ability of these instruments to capture different RRB constructs is needed.

RRB is an umbrella term for a set of heterogeneous behaviors that include motor stereotypies, daily routines and rituals, repetitive play, preoccupations with particular interests and compulsive activities related to these preoccupations. Given this complexity and heterogeneity, RRB are best understood as a multidimensional construct with a range of distinct behavioral domains [4, 15–17]. A variety of approaches have been taken to organize and define RRB in ASD. Approach based on clinical and developmental considerations, initiated by Prior and Macmillan [16] and significantly expanded by Turner [17] suggested that RRB can be divided into broad ‘lower-order’ and ‘higher-order’ domains based on their pattern of emergence during normative development and the relationship with IQ/cognitive functioning. Our current understanding of RRB in ASD has been influenced by the factor analytic explorations of the currently available dedicated RRB measures including the Repetitive Behavior Scale-Revised (RBS-R [18]), the Repetitive Behavior Questionnaire (RBQ [19]), the Repetitive Behavior Questionnaire-2 (RBQ-2 [20–22]), the Childhood Routines Inventory (CRI [23, 24]) and the Children's Yale-Brown Obsessive Compulsive Scale (CY-BOCS [25]). Initial factor analytic work suggested two factor-solution encompassing Repetitive Motor Behaviors (RMB) domain which included behaviors such as hand and finger mannerisms, stereotyped body movements, and repetitive manipulation of objects or parts of objects, and Insistence on Sameness (IS) domain which included behaviors such as ritualistic behaviors and resistance to changes in routine or personal environment [26, 27]. However, studies by Leekam and colleagues [22], Lam, Bodfish and Piven [11] and Honey et al. [15] highlighted unusual/circumscribed interests (CI) with over-focus on specific topics, objects, stimuli and/or activities as an additional domain. This three-factor structure has been largely replicated across subsequent studies (e.g., [8]).

To evaluate the validity of RMB, IS and CI, several studies have explored relationships of these three RRB domains with age, cognitive ability and co-occurring symptoms such as anxiety. For instance, RMB tend to be related to younger age and lower IQ [8, 26] and are relatively independent of anxiety levels [11]. By contrast, some, but not all studies, have observed associations between IS and CI with older age and higher IQ [5, 28, 29]. IS has been consistently associated with more severe anxiety [11, 30–33]. Preliminary evidence also suggests that RMB, IS and CI domains have distinct neural [34] and genetic underpinnings [35–38] and show different familial patterns [10, 39, 40, 41].

Despite the mounting evidence suggesting the multidimensionality of the RRB, the factor structure of this domain captured by widely used screening, diagnostic and quantitative severity ASD measures remains unexplored. The only exception is the Autism Diagnostic Interview-Revised (ADI-R [42]) which has been evaluated in several studies [8, 28, 37]. However, although RMB, IS and CI factors seem to provide a relatively stable explanation of the ADI-R RRB factor structure, the actual item content of these factors has varied considerably across investigations. For instance, depending on the study, unusual attachment to objects and unusual preoccupations items have loaded onto RMB [8, 28, 37], IS [43] and CI [10, 15] factors or were not included in the final factor solution [26, 30].

Although diagnostic and quantitative ASD measures such as the SRS-2, the SCQ, the ADI-R and the ADOS have not been specifically designed to provide an in-depth, fine-grained assessment of distinct RRB domains such as RMB, IS and CI, these instruments are widely used across ASD research and clinical practice and have been included in several large datasets that are available to the research community (e.g., Simons Simplex Collection, Autism Genetic Resource Exchange, Autism Brain Imaging Data Exchange). Establishing the ability of noted measures to capture distinct RRB domains, even in a manner that is somewhat suboptimal when compared to dedicated instruments such as the RBS-R, the RBQ-2, the CRI or the CY-BOCS, can provide a valuable resource for RRB-related investigations across both clinical and research contexts. Therefore, the present study aimed to capitalize on the availability of large, publicly available databases to conduct a stringent evaluation of the RRB factor structure across the SRS-2, the SCQ, the ADI-R and the ADOS. We predicted that RMB, IS and CI factors would emerge across the majority of measures, with a potential exception of the ADOS due to the limited range of items. Given the focus of measures developed in the context of ASD and utilization of existing sample, derived findings specifically speak to the RRB structure in ASD. Future studies utilizing samples that span normative and atypical development and measures sampling RRB symptoms and domains that occur across a range of clinical populations and typical early development are needed to derive more general, transdiagnostic RRB structure. In addition to investigating RRB factor structure across the SRS-2, SCQ, ADI-R and ADOS, an important further step was to appraise the validity of the derived factors by exploring their convergence with a well-established, dedicated RRB measure—the RBS-R and the pattern of relationships with external validators such as age, sex, cognitive functioning, anxiety and disruptive behaviors. Crucially, despite the potential short-term utility of the factors derived here, the longer-term goal of the field should be to utilize quantitative instruments specifically developed to comprehensively capture distinct RRB domains. Therefore, an additional aim of this analysis was to derive information necessary for future measurement development.

## Methods

### Research participants

Data were obtained from five publicly available databases: Healthy Brain Network (HBN [44]); National Database for Autism Research (NDAR; https://ndar.nih.gov); Simons Simplex Collection (SSC [45]); Autism Genetic Research Exchange (AGRE [46]); and Interactive Autism Research Database (IAN; http://iancommunity.org). Only individuals with item-level data and with a diagnosis of ASD were included in this study. All participants or their parent/legal guardians have provided informed consent for participation as part of the original investigations. For the SRS-2, the final sample consisted of N = 16,761 individuals (*M*_*age*_ = 9.43, *SD* = 3.73; 18.5% female); for the SCQ, N = 15,840 (*M*_*age*_ = 7.99, *SD* = 4.06; 18.1% female); for the ADI-R, N = 8985 (*M*_*age*_ = 8.86, *SD* = 4.68; 19.4% female); and for the ADOS modules 3 and 4, of N = 6314 (*M*_*age*_ = 12.29, *SD* = 6.79; 17.7% female). Table 1 presents demographic characteristics for each of the measures broken down by each database.

**Table 1:** Demographic characteristics of youth participants in publicly available data sets

	Study				
	AGRE	NDAR	SSC	IAN	HBN
SRS-2	N = 2282	N = 3365	N = 2867	N = 8132	N = 115
Mean age (SD), years	8.34 (3.54)	9.48 (3.55)	9.03 (3.56)	9.75 (3.85)	10.31 (2.99)
Female %	31.4	17.8	13.6	18.1	19.6
SCQ	–	N = 1012	N = 1185	N = 13,541	N = 102
Mean age (SD), years	–	5.94 (4.04)	8.82 (3.62)	8.05 (4.05)	10.31 (2.99)
Female %	–		14.3	18.4	19.6
ADI-R	N = 3733	N = 2398	N = 2854	**–**	**–**
Mean age (SD), years	8.73 (4.86)	8.89 (5.46)	9.02 (3.57)	–	–
Female %	22.5	21.2	13.6	–	–
ADOS Module 3	N = 1159	N = 2330	N = 1629	**–**	**–**
Mean age (SD), years	9.52 (3.0)	10.34 (3.19)	9.75 (3.15)	–	–
Female %	17.9	24.3	11.3	–	–
ADOS Module 4	N = 264	N = 849	N = 83	**–**	**–**
Mean age (SD), years	18.89 (7.52)	23.69 (9.24)	16.26 (1.40)	–	–
Female %	28.4	17.6	10.8	–	–

ADI-R, Autism Diagnostic Interview Revised; ADOS, Autism Diagnostic Observation Schedule; AGRE, Autism Genetic Research Exchange; NDAR, National Database for Autism Research; SCQ, Social Communication Questionnaire; SRS, Social Responsiveness Scale standardization dataset; SSC, Simons Simplex Collection; IAN, Interactive Autism Research Database; HBN, Healthy Brain Network

### Measures

The Social Responsiveness Scale (SRS [12]) is a 65-item measure designed to index severity in social impairments and the presence of RRB. Each item is rated on a 4-point scale (from 1 = Not True to 4 = Almost Always True) with higher scores indicating higher trait severity/atypicality.

The Social Communication Questionnaire (SCQ [13]) is a 40-item parent-report questionnaire designed to index the severity of impairments in social, communication and RRB domains seen in ASD. Each item is scored using the dichotomous response format, with a value of one indicating the presence of atypicality and a value of zero the absence of atypicality. In this investigation, we have focused on the lifetime ratings.

The Autism Diagnostic Interview-Revised (ADI-R [42]) is a semi-structured parent interview informed by the ICD-10 and DSM-IV intended to assist with the diagnosis of ASD. In this investigation, we have primarily focused on the current scores; however, supplemental analysis of the ever scores was also conducted and is included in Additional file 1: Table S1.

The Autism Diagnostic Observation Schedule (ADOS [14]) is a semi-structured instrument that allows direct observation of children during specific play, social and language tasks. The ADOS consists of four modules based on the chronological age and level of expressive language. Modules 1 and 2 were not considered in this analysis given that one of the items (D4) at the same time assesses IS, CI and repetitive manipulation of objects. Further, Modules 3 and 4 have an additional item that captures routines and ritualistic behaviors (item D5). Therefore, while not ideal, Modules 3 and 4 present with higher opportunity to derive distinct, fine-grained factors.

The Repetitive Behaviors Scale-Revised (RBS-R [18]) is a 43 item parent-report measure designed to capture a wide range of RRB. Although it was originally proposed that the RBS-R items constitute six distinct subscales, subsequent factor analyses mostly failed to support the original 6-factor solution. A five-factor solution derived by Bishop and colleagues [8] was utilized here with the Stereotypy factor corresponding to RMB, the Ritualistic/Sameness factor corresponding to IS and the Restricted Interests factor corresponding to CI.

For each of the measures, items were selected based on the manual. In addition, given the sometimes unclear boundaries between RRB and social communication symptoms (e.g., talking excessively about topics of special interest), the first and last author reviewed individual items across the measures to ensure that any potentially relevant items were not missed. Further, where possible, item selection was guided by the previous factor analytic investigations. Given that the SRS-2 has only one item relating to the sensory sensitivity (item 42: seems overly sensitive to sounds, textures, or smiles) and single-item factor is not viable, this item was not included in the analysis. ADI-R has two items that relate to the sensory sensitivity—item 72 (undue general sensitivity to noise) and 73 (abnormal response to specific sensory stimuli) that have been included in only a few of the previous ADI-R factor analyses [8, 15, 27, 47]. Curiously, across all noted studies, items 72 and 73 have loaded onto the IS factor rather than forming a separate sensory sensitivity factor which is most likely due to a combination of the fact that two-item factors are difficult to extract, especially with limited sample sizes, and the fact that sensory sensitivity is highly associated with anxiety [11] which is, in turn, a major driver behind IS [30, 33]. Therefore, we have run a preliminary analysis with items 72 and 73 included and they have loaded onto the IS factor rather than forming separate construct, consistent with noted previous factor analyses. Given the loading onto the IS factor, we have omitted these two items from the models described below. The rationale for excluding these two items was related to the fact that sensory sensitivity and IS are clearly distinct constructs and mixed IS factor could make it more difficult for future studies to interpret the results (e.g., it would not be clear whether association with a particular clinical, genetic or neural correlate is driven by IS or sensory sensitivity aspect). Finally, although ADI-R item 79 (midline hand stereotypies) was not included in the previous studies, we have decided to include item 79 given that this particular RRB does occur in ASD and also occurs (more frequently) across several genetic disorders where ASD is highly prevalent.

The following items were included in the analysis: *SRS-2:* items 4 (inflexible/rigid behaviors), 20 (unusual sensory interests), 24 (difficulties with changes in routine), 28 (thinks about the same thing), 31 (fixated on certain topics/thoughts), 39 (narrow range of interests), 50 (motor mannerisms) and 61 (inflexible); *SCQ:* items 7 (says the same thing over and over/insists that someone else says the same thing over and over), 8 (has rituals or has to do things in a particular way or order/insists that others go through said rituals), 11 (unusual interests [e.g., traffic lights]), 12 (interest in parts of a toy/object), 13 (interests unusual in terms of intensity but typical in terms of content), 14 (sensory interests), 15 (motor mannerisms), 16 (complex body movements) and 18 (toy and object that she/he carries around); *ADI-R:* items 39 (verbal rituals), 67 (unusual preoccupations), 68 (circumscribed interests), 69 (repetitive object use/interest in parts of objects), 70 (compulsions/rituals), 71 (unusual sensory interests), 74 (difficulties with changes in own routines and/or environment), 75 (resistance to trivial changes in the environment not related to the individual), 76 (unusual attachment to objects), 77 (hand and finger mannerisms), 78 (complex mannerisms) and 79 (midline hand movements); *ADOS (modules 3 and 4):* items D1 (unusual sensory interest in play material/person), D2 (hand a finger and other complex mannerisms), D4 (described above) and D5 (compulsions or rituals).

### Data analysis

For each of the measures, item-level data were combined across studies and the total sample was randomly split into exploratory and validation subsamples (SRS-2: Exploratory: *N* = 8409, Validation: *N* = 8352; SCQ: Exploratory: *N* = 8054, Validation: *N* = 7786; ADI-R: Exploratory: *N* = 4518, Validation: *N* = 4467; ADOS: Exploratory: *N* = 3180, Validation: *N* = 3134). Exploratory and validation samples did not differ in age and sex distribution. In order to derive factor structure of the RRB domain, a series of latent variable analyses were conducted separately for each of the measures using steps described henceforth. Exploratory application of the Exploratory Structural Equation Modeling (ESEM [48, 49]) used geomin rotation (given that constructs are likely to be correlated [49]). Given the number of items and number of factors derived in previous factor analytic studies across a range of dedicated RRB and general ASD severity measures, models with 1–5 factors were estimated. The number of factors to be retained was guided by a range of recommended fit indices [49–53]; the Comparative Fit Index (CFI), the Tucker-Lewis Index (TLI), the Root Mean Square Error of Approximation (RMSEA) and the standardized root mean square residual (SRMR). The following cut-offs were applied: (i) CFI and TLI values > .90 indicating adequate and > .95 excellent fit; (ii) RMSEA and SRMR values of < .08 indicating adequate and < .06 excellent fit, with the close fit-test with a *p* value > .05. Because chi-square index tends to be oversensitive to a range of factors including sample size, it was not used. Analyses used polychoric correlations with the weighted least square estimator. Models derived through exploratory models were replicated in the validation sample using the confirmatory application of the ESEM. Model fit was evaluated using noted fit indices. The derived factor solutions were further replicated across sex and age (samples were divided into subsamples aged 2–6, 7–12 and 13 + years). These analyses were run using MPlus [54].

The relationship between derived latent RRB subscales and several variables of interest including chronological age, sex, verbal (VIQ) and non-verbal IQ (NVIQ) and with the Child Behavior Checklist (CBCL [55]) was explored using Pearson’s correlation coefficient and one-way analysis of variance (ANOVA). All correlations and comparisons were conducted in SPSS [56] and performed through bootstrapping using 5,000 resamples to provide more robust statistics and account for the potential skewness of the data [57]. All comparisons were supplemented with the effect sizes.

## Results

The exploratory (referred to as EFA in Table 2) and confirmatory ESEM (referred to as ESEM in Table 2) models are presented in Table 2. As can be seen, across all of the measures, unidimensional models had unsatisfactory fit.

**Table 2:** Summary of goodness of fit statistics for the tested factor analysis models

Model		χ^2^	CFI	TLI	RMSEA	SRMR
**SCQ**						
EFA Unidimensional	Sample 1	577.668**	.903	.871	.071**	.069
EFA 2-Factor	Sample 1	132.470**	.980	.962	.039**	.030
EFA 3-Factor	Sample 1	16.176	.999	.998	.009	.011
ESEM 3-Factor	Sample 2	34.903**	.995	.986	.022	.016
	Female Sex/gender	17.098	.997	.992	.017	.017
	Male Sex/gender	29.386*	.998	.994	.015	.011
	Age group 1^a^	42.884**	.997	.990	.019	.013
	Age group 2^b^	43.648*	.996	.988	.021	.015
	Age group 3^c^	30.61**	.995	.984	.026	.020
**SRS-2**						
EFA Unidimensional	Sample 1	3796.694**	.825	.754	.150**	.070
EFA 2-Factor	Sample 1	1374.531**	.937	.864	.112**	.034
EFA 3-Factor	Sample 1	23.619**	.999	.997	.017	.004
ESEM 3-Factor	Sample 2	21.284**	.999	.997	.016	.004
	Female Sex/gender	27.201**	.998	.992	.031	.006
	Male Sex/gender	28.311**	.999	.997	.015	.003
	Age group 1^a^	13.131	.999	.998	.014	.004
	Age group 2^b^	48.439**	.999	.995	.027	.005
	Age group 3^c^	3.44	.999	.998	.008	.003
**ADI-R**						
Current scores						
EFA Unidimensional	Sample 1	1951.781**	.708	.643	.091**	.086
EFA 2-Factor	Sample 1	219.452**	.973	.958	.031	.029
EFA 3-Factor	Sample 1	142.231**	.983	.966	.028	.024
ESEM 3-Factor	Sample 2	84.499**	.991	.980	.024	.003
	Female Sex/gender	36.983	.996	.991	.017	.020
	Male Sex/gender	137.23**	.988	.974	.026	.019
	Age group 1^a^	107.986**	.983	.962	.031	.023
	Age group 2^b^	32.346	.997	.994	.013	.019
	Age group 3^c^	28.838	.998	.996	.011	.020
ADOS Modules 3 and 4						
EFA unidimensional	Sample 1	44.137**	.915	.756	.083	.035
	Sample 2	42.256**	.930	.780	.082	.035

ADI-R, Autism Diagnostic Interview-Revised; CFA, Confirmatory Factor; CFI, Comparative Fit Index; ESEM, Exploratory Structural Equation Modeling; RMSEA, Root Mean Square Error of Approximation; SCQ, Social Communication Questionnaire; SRMR, Standardized Root Mean Square Residual; SRS-2, Social Responsiveness Scale; TLI, Tucker-Lewis Index

*p < .01; **p < .001

^a^Age Group 1: 2–6 years; ^b^Age Group 2: 7–12 years; ^c^Age Group 3: 13 years and above

**Fig. 1:**
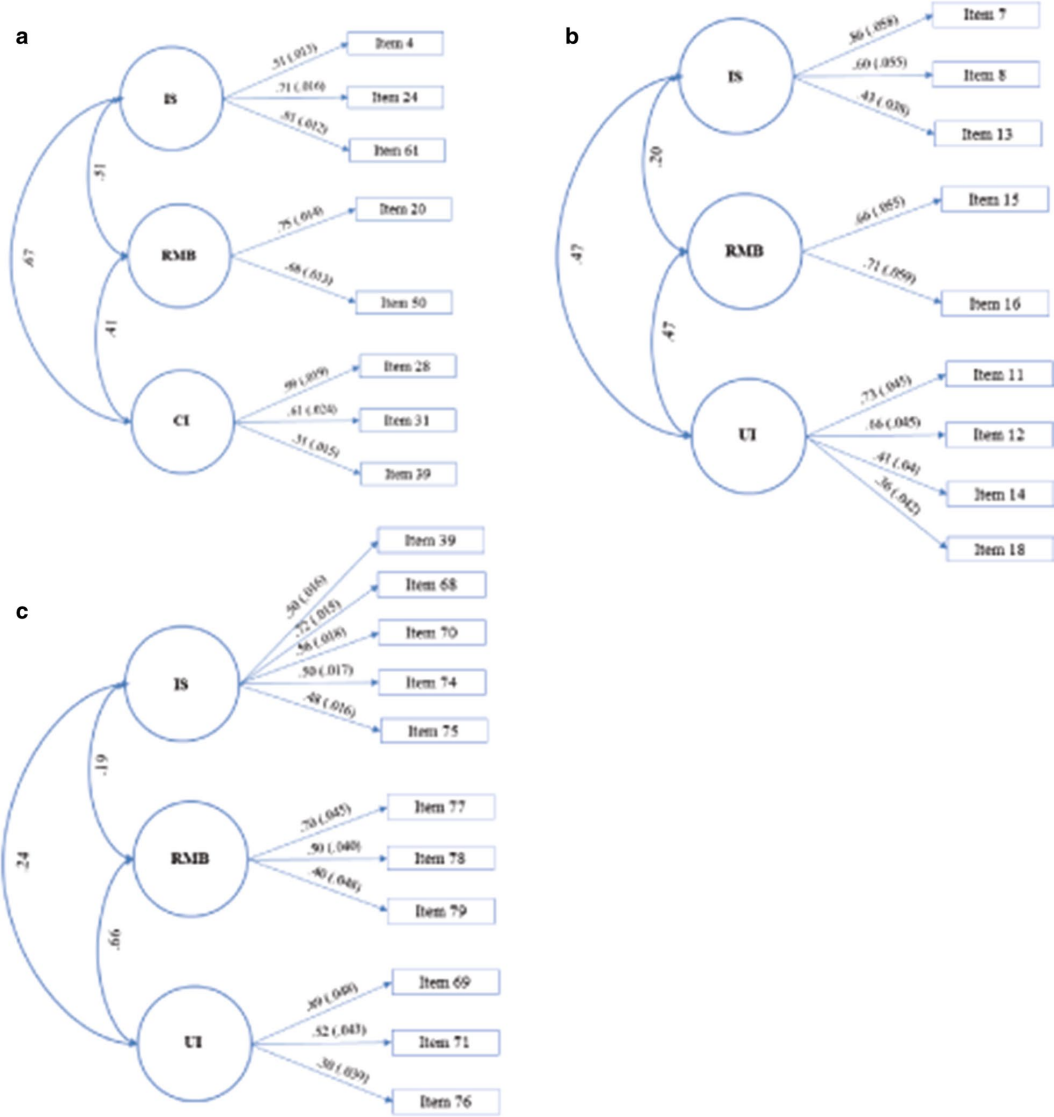
**a** Exploratory Structural Equation Modeling Social Responsiveness Scale correlated 3-factor RRB solution. Solid lines represent factor loadings, and curved lines represent the correlation among factors. *Note*: CI: Circumscribed Interests; IS: Insistence on Sameness; RMB: Repetitive Motor Behaviors. Item 4 (Shows rigid pattern of behaviors when under stress), item 20 (Unusual sensory interests), item 24 (Difficulties with changes in routine), item 28 (talks about the same thing or topic), item 31 (cannot get his mind of something), item 39 (Unusually narrow range of interests), item 50 (Repetitive motor behaviors such as hand flapping), item 61 (Inflexible), **b** Exploratory Structural Equation Modeling Social Communication Questionnaire correlated 3-factor RRB solution. Solid lines represent factor loadings, and curved lines represent the correlation among factors. *Note*: IS: insistence on sameness; RMB: repetitive motor behaviors; UI: unusual interests. Item 7 (Says the same thing/insists that others say the same thing over and over), item 8 (Has to do things in a very particular order), item 11 (Interests odd in terms of content [e. g., traffic lights, timetables]), item 12 (Interest in parts of objects [e. g., spinning wheels]), item 13 (interests unusual in intensity but not content/topic), item 14 (unusually interested in sensory stimuli), item 15 (Motor mannerisms/stereotypies [e.g., hand flapping, finger movements]), item 16 (Complex body movements [e.g., spinning, bouncing up and down]), item 18 (Objects to carry around). **c** Exploratory Structural Equation Modeling Autism Diagnostic Interview-Revised correlated 3-factor RRB solution. Solid lines represent factor loadings, and curved lines represent the correlation among factors. *Note*: IS: insistence on sameness; RMB: repetitive motor behaviors; UI: unusual interests. Item 39 (Verbal rituals), item 68 (Circumscribed interests), item 69 (Repetitive use of/interest in parts of objects), item 70 (Compulsions/rituals), item 71 (Unusual sensory interests), item 74 (Difficulties with minor changes in one’s own routines/environment), item 75 (Resistance to changes in the environment not directly affecting the individual), item 76 (unusual attachment to objects), item 77 (Hand and finger mannerisms), item 78 (Complex mannerisms or body movements), item 79 (Midline hand movements)

**SRS-2**: The three-factor model had a superior fit to the two-factor model (CFI = .999, TLI = .997, RMSEA = .017, SRMR = .004 vs CFI = .937, TLI = .864, RMSEA = .112, SRMR = .034) and the four-factor model failed to converge. Thus, the three-factor model was retained. Factors were interpreted as repetitive motor behaviors (RMB; 2 items), insistence on sameness (IS; 3 items) and circumscribed interests (CI; 3 items). Confirmatory application of the ESEM demonstrated excellent fit of this model in the validation sample (CFI = .999, TLI = .997, RMSEA = .016, SRMR = .004) and showed excellent fit across sex and age (all CFI ≥ .998, all TLI ≥ .992, all RMSEA ≤ .031, all SRMR ≤ .006). Figure 1a shows factor loadings, standard errors (SE) for each of the loadings (SE) and factor correlations for the confirmatory ESEM for the whole sample. Item loadings for the exploratory three-factor model are provided in Additional file 1: Table S2.

**SCQ**: The three-factor model had a superior fit to the two-factor model (CFI = .999, TLI = .998, RMSEA = .009, SRMR = .011 vs CFI = .980, TLI = .962, RMSEA = .039, SRMR = .030) and the four-factor model failed to converge. Although the two- and three-factor solutions had excellent fit indices, the three-factor solution was retained because additional meaningful factor emerged. Factors were interpreted as repetitive motor behaviors (RMB; 2 items), insistence on sameness (IS; 3 items) and unusual interests (UI; 4 items). The unusual interest factor included sensory interests (item 14), interest in objects/part of objects (item 12) and interests with a narrow focus (e. g. traffic lights or train tables) (item 11). Item 12 (interest in objects/part of objects) loaded on IS and UI factors but was assigned to the IS factor given the higher loading (.44 vs .34). Confirmatory application of the ESEM showed excellent fit of the three-factor model in the validation sample (CFI = .995, TLI = .986, RMSEA = .022, SRMR = .016) and excellent fit across sex and age subgroups (all CFI ≥ .995, all TLI ≥ .985, all RMSEA ≤ .025, all SRMR ≤ .020). Factor loadings, SE and correlations for the confirmatory ESEM are shown in Fig. 1b. Item loadings for the exploratory three-factor model are provided in Additional file 1: Table S3.

**ADI-R:** Two- and three-factor solutions provided excellent fit (CFI = .973, TLI = .958, RMSEA = .031, SRMR = .029 vs CFI = .983, TLI = .966, RMSEA = .028, SRMR = .024) and four-factor solution failed to converge. In the two-factor solution, RMB and unusual/circumscribed interests items loaded on a single factor. Because RMB and UI emerged as separate factors, the three-factor solution, including RMB, IS and UI, was retained. Three-factor solution was replicated in the validation sample (CFI = .991, TLI = .980, RMSEA = .024, SRMR = .019). The fit across sex and age was excellent (all CFI ≥ .988, all TLI ≥ .973, all RMSEA ≤ .27, all SRMR ≤ .020). Figure 1c shows factor loadings, SE and correlations for the three-factor solution. Item loadings for exploratory three-factor model are provided in Additional file 1: Table S4. Additional file 1: Table S1 shows that three-factor solution provided the best fit for ADI-R ever scores (exploratory sample: CFI = .983, TLI = .966, RMSEA = .026, SRMR = .021; validation sample: CFI = .988, TLI = .973, RMSEA = .026, SRMR = .020).

**ADOS Modules 3 and 4:** As noted, the unidimensional model had a poor fit in the exploratory sample, however, the two-factor model failed to converge. The unidimensional and two-factor models were conducted in the validation sample with unidimensional model showing poor fit (CFI = .930, TLI = .789, RMSEA = .082, SRMR = .035) and two-factor model failing to converge.

**Cross-measure ESEM:** we have utilized the cross-measure exploratory ESEM to combine the SRS-2 and ADI-R items to investigate whether additional RRB factors would emerge. Given that the similarity between the ADI-R and the SCQ, the dichotomous nature of the SCQ scoring and that the available SCQ data were from the lifetime rather than the current form, the analysis focused on the SRS-2 and ADI-R. Where identical items existed across the SRS-2 and ADI-R, only one of the items was included to avoid problems with the convergence and fit. Finally, SRS-2 item 42 and ADI-R items 72 and 73 capturing different aspects of sensory sensitivity were included in the analysis. Three-factor solution showed adequate fit (CFI = .949, TLI = .923, RMSEA = .049, SRMR = .039) and four- (CFI = .979, TLI = .959, RMSEA = .036, SRMR = .029) and five-factor (CFI = .992, TLI = .984, RMSEA = .023, SRMR = .021) solutions showed excellent fit. Factor loadings for four- and five-factor solutions are showed in Additional file 1: Tables S5 and S6. RMB, IS, UI and CI factors emerged across both four- and five-solutions; the main distinction was that while in the four-factor solution three sensory sensitivity items (SRS-2 item 42 and ADI-R items 72 and 73) loaded onto the IS factor, in the five-factor solution these items formed a separate sensory sensitivity factor. Therefore, the five-factor solution was considered more optimal.

Table 3 shows the patterns of associations between the SRS-2, the SCQ and the ADI-R RRB scores and corresponding RBS-R subscales scores. RBS-R data were available for N = 4097 participants with the SRS-2, N = 3489 with the ADI-R and N = 1602 with the SCQ data. Using Fisher r-to-z transformation on the Pearson correlation values, we examined the convergent and divergent validity of the RMB, IS CI and UI scores across the SRS-2, the SCQ and the ADI-R. The association between RMB scores with the RBS-R Stereotypy subscale was significantly stronger when compared to other subscales in SRS-2 (Fisher r-to-z Z range: 14.36–23.83, all *p* < .001), in SCQ (Z range: 6.69–11.56, all *p* < .001), and in ADI-R (Z range: 11.41–16.59, all *p* < .001). Significantly stronger associations were observed between IS scores with the RBS-R Ritualistic/Sameness subscale when compared to other subscales in SRS-2 (Fisher r-to-z Z range: 12.52–18.08, all *p* < .001), in SCQ (Z range: 1.79, *p* = .037–6.98, *p* < .001), and ADI-R (Z range: 5.46–11.72, all *p* < .001). SRS-2 CI score showed a significantly stronger association with the RBS-R Restricted subscale when compared to the Stereotypy subscale (Fisher r-to-z Z range: 11.40, *p* < .001) but similar strength of the relationship as with the RBS-R Ritualistic/Sameness subscale. UI subscale of both the SCQ and the ADI-R showed significantly higher associations with the RBS-R Restricted Behaviors subscale than with the RBS-R Ritualistic/Sameness subscale (SCQ: Fisher r-to-z Z = 5.34, *p* < .001; ADI-R: Z = 4.69, *p* < .001); however, no differences were observed in terms of association with the Stereotypy subscale.

**Table 3:** Pattern of associations between identified RRB factors with other individual characteristics

	Age	NVIQ	VIQ	CBCL anxiety	CBCL externalizing	RBS-R stereotypy	RBS-R ritualistic/sameness	RBS-R restricted behavior
SRS-2 RMB	− .102**	− .359**	− .329**	.135**	.229**	.644**	.272**	.38**
SRS-2 IS	.052**	.024	.06**	.402**	.48**	.237**	.558**	.34**
SRS-2 CI	.151**	− .014	.043**	.319**	.276**	.194**	.435**	.42**
SCQ RMB	− .07**	− .346**	− .374**	− .07	.004	.382**	.005	.09**
SCQ IS	.137**	− .038	− .052	.02	.088**	.021	.262**	.18**
SCQ UI	.01	− .328**	− .338**	− .05	.100**	.277**	.092**	.27**
ADI-R RMB	− .166**	− .212**	− .202**	.001	.03	.444**	.133**	.16**
ADI-R IS	.160**	.065**	.066**	.084**	.105**	.071**	.337**	.18**
ADI-R UI	− .236**	− .299**	− .301**	.026	.163**	.390**	.222**	.33**

ADI-R, Autism Diagnostic Interview-Revised; CBCL, Child Behavior Checklist; CI, Circumscribed Interests; IS, Insistence on Sameness; NVIQ, Non-verbal IQ; RBS-R, Repetitive Behavior Scale-Revised; RMB, Repetitive Motor Behaviors; SCQ, Social Communication Questionnaire; SRS-2, Social Responsiveness Scale; VIQ, Verbal IQ; UI, Unusual Interests

*p < .01; **p < .001

As can be seen from Table 3, younger age was associated with higher RMB and lower IS factors across all scales; however, although significant, associations were weak. IQ data were available for 2742 participants with the SRS-2, for 2755 participants with the ADI-R, and for 1178 participants with the SCQ. The CBCL data were available for 3532 participants with the SRS-2, for 3402 participants with the ADI-R, and for 1240 participants with the SCQ. Verbal (VIQ) and non-verbal IQ (NVIQ) scores were inversely correlated with RMB across all measures and with UI on SCQ and ADI-R. The associations between VIQ and NVIQ with IS were of low magnitude. CBCL anxiety scores were positively associated with the SRS-2 and ADI-R IS scores as well as with SRS-2 CI and RMB scores. The IS-anxiety correlation between SRS-2 IS (*r* = .40) was significantly higher than RMB-anxiety correlation (*r* = .13); Fisher r-to-z Z = 12, *p* < .001. CBCL externalizing scores were positively associated with IS and CI scores across all measures and with the RMB scores on the SRS-2 (but not on SCQ and ADI-R). Mean scores were significantly higher in males compared to female participants across all RRB subscales (Table 4).

**Table 4:** Sex comparison across RRB factors

	Male Mean (SD)	Female Mean (SD)	Statistics
SRS-2 RMB	2.80 (1.97)	2.42 (2.05)	*F* = 91. 87, *p* < .001, *ƞ*^2^ = .006
SRS-2 IS	5.35 (2.45)	4.88 (2.68)	*F* = 88.90, *p* < .001, *ƞ*^2^ = .005
SRS-2 CI	5.70 (2.39)	4.87 (2.68)	*F* = 282.84, *p* < .001, *ƞ*^2^ = .017
SCQ RMB	1.40 (.75)	1.35 (.76)	*F* = 8.76, *p* < .001, *ƞ*^2^ = .001
SCQ IS	2.12 (.94)	2.04 (.96)	*F* = 17.87, *p* < .001, *ƞ*^2^ = .001
SCQ UI	2.61 (1.20)	2.46 (1.27)	*F* = 38.26, *p* < .001, *ƞ*^2^ = .002
ADI-R RMB	1.72 (1.54)	1.51 (1.49)	*F* = 25. 91, *p* < .001, *ƞ*^2^ = .003
ADI-R IS	3.20 (2.37)	2.79 (2.39)	*F* = 40. 57, *p* < .001, *ƞ*^2^ = .005
ADI-R UI	2.20 (1.60)	2 (1.61)	*F* = 20. 21, *p* < .001, *ƞ*^2^ = .002

ADI-R, Autism Diagnostic Interview-Revised; CBCL, Child Behavior Checklist; CI, Circumscribed Interests; IS, Insistence on Sameness; RMB, Repetitive Motor Behaviors; SCQ, Social Communication Questionnaire; SRS-2, Social Responsiveness Scale; UI, Unusual Interests

## Discussion

The current study utilized large, publicly available databases to explore the capacity of the Social Responsiveness Scale (SRS-2), the Social Communication Questionnaire (SCQ), the Autism Diagnostic Interview-Revised (ADI-R) and the Autism Diagnostic Observation Schedule (ADOS) to capture distinct constructs within the restricted and repetitive behaviors (RRB) domain in autism spectrum disorder (ASD). With the exception of the ADOS, a three-factor structure emerged as the best fit to data for the other three measures. The three-factor model was confirmed in the second round of analyses and performed well across sex and age. Repetitive-motor behaviors (RMB) and insistence on sameness (IS) factors were consistently identified. However, the domain of circumscribed interests (CI) and unusual interests (UI) was not uniform. The SRS-2 recognized circumscribed interests with items capturing interests unusual in terms of their focus, intensity and inflexibility but typical/age-appropriate in terms of their content/subject (e.g., particular animals, fictional characters or content topic in children). By contrast, the unusual interests factor encompassing items such as fascination with sensory stimuli, fascination with parts of the object and interests unusual in terms of their content/subject (timetables, traffic lights) emerged from the SCQ and ADI-R. Given the relationship between RMB and UI factors, it will be important for future studies to further evaluate their distinctiveness Analyses of ADOS modules 3 and 4 suggest that more fine-grained RRB models are not viable.

Content of RMB and IS factors identified across the SRS-2, SCQ and ADI-R conceptually resemble the content within cognate subscales of the RBS-R as well as other instruments including the RBQ, the RBQ-2, and the CRI-R. For example, the RMB and IS factors derived here showed significantly stronger associations with the corresponding RBS-R subscales (for instance, RMB subscale was associated more strongly with RBS-R Stereotypy subscale than with RBS-R Ritualistic/Sameness and Restricted subscales). Furthermore, ADI-R RMB factor derived here encompassed items 77 and 78 that consistently loaded on RMB factor across a range of previous ADI-R factor analytic explorations (e.g., [8, 10, 26, 28, 40]). RMB factor derived in our study further included item 79 that was not been included in previous ADI-R investigations. With the exception of sensory hyper-sensitivity items, ADI-R IS factor identified in this study resembled the IS factor derived in several previous ADI-R factor analyses [8, 10, 26, 28]. Repetitive use/interest in parts of objects (item 69) and unusual sensory interests items (item 71) that loaded onto the RMB factor across several previous ADI-R studies [8, 26, 28] loaded onto a separate UI ADI-R factor in our investigation. This was most likely due to the large sample size enabling more fine-grained factors to emerge. UI factor derived in our analysis bears a strong resemblance to the ADI-R Sensory Interests factor derived by Frazier and Hardan [2017] and to the complex stereotypies factor derived by Smith and colleagues [43]. In addition, UI factor derived in our study resembled the Unusual Sensory Interests factor from the RBQ-2 identified by Leekam et al. (2007). ADI-R item 68 that specifically refers to interests that are unusual in terms of their inflexibility has loaded onto the IS factor in our study. This finding is consistent with several previous ADI-R factor analyses (e.g., [8, 38, 43, 47]) and most likely due to the fact that lack of flexibility is a common element across IS and this CI subtype. If additional ADI-R items capturing CI subtype that is characterized by inflexibility but typical content were available, it is likely that they would load onto separate factors, as was the case with the SRS-2. This was indeed confirmed by the findings from the cross-measure ESEM.

The secondary aim of the current study was to characterize the relationship between derived RRB factors with other individual characteristics. Associations with age, cognitive functioning, anxiety and externalizing symptoms suggested somewhat distinctive patterns. Higher RMB and UI were associated with younger age and lower VIQ and NVIQ. IS scores across all measures and CI scores on the SRS-2 were directly correlated with older age and to a lesser degree but in the same direction with VIQ and NVIQ. Applying Fisher’s r to z transformation anxiety was more strongly associated with IS and CI factors across the ADI-R and SRS-2, and RBS-R than with RMB subscale across these measures. This observation is consistent with prior studies from our group [11, 33, 59, 60] and others [30–32]. The association of anxiety with IS implies that successful treatment of anxiety might also benefit IS [33]. Externalizing symptoms were positively correlated with the SRS-2 subscales, especially the IS subscale. The correlations of externalizing symptoms with subscales on all other measures were weakly positive. Finally, the examination of sex differences showed that mean scores on all RRB subscales were greater across all measures in males compared to females.

The negative association between RMB with age and IQ identified aligns with Turner’s [17] conceptualization of lower-order RRB and with findings from longitudinal and cross-sectional studies in ASD [8, 26, 58] and community samples [61]. The positive relationship between age with IS and CI (as measured by the SRS-2) and weaker relationship with IQ is also in line with a range of previous studies [5, 10, 29]. The observed association between anxiety and more severe IS is supported by several previous studies [11, 30, 33, 59, 60]. Moreover, the positive association between anxiety and externalizing problems with IS reported here is also consistent with other studies [31, 62]. In normative development, emergence and increase in IS occur along with the emergence of normative fears [63]. This suggests that IS may serve as an early form of self-regulation by exerting control on the environment, limiting unpredictability, and reducing normative fears [33, 64, 65]. With the development of more mature forms of self-regulation, IS is reduced and therefore transitory [61]. However, given the impairments in self-regulation in ASD, children with ASD may continue to rely on IS to manage anxiety [33, 61]. Reliance on IS, ironically, does not appear to be effective in the long-term and might even exacerbate anxiety [33]. In addition, it has been suggested that over-reliance on IS might further compromise the development of self-regulation due to the restrictive nature of IS and limited opportunities for learning and development [33]. Furthermore, deviations from routines can exacerbate anxiety and lead to meltdowns and destructive and aggressive behavioral patterns, which is in line with the positive association between IS and externalizing problems reported here. The relationship between CI with anxiety and externalizing problems found in our study is likely to be a consequence of individuals not being able to engage with their preferred interests and activities. For instance, one of the common themes that emerged from a qualitative study by Halim et al. [62] that investigated presentation and triggers for anxiety in ASD was that prevention of engaging in both preferred interests and routines could be a cause of anxiety, stress and meltdowns. Although findings reported here provide further support to the previously suggested link between IS and CI with internalizing and externalizing problems, and the potential mediating and/or moderating role of impairments in self-regulation, these findings are based on cross-sectional and correlational findings. Therefore, future research using longitudinal designs, especially focusing on early stages of development when these behaviors start to emerge, is needed to disentangle the nature of the reported relationships and elucidate whether these symptom domains are mechanistically linked (e. g. elevated anxiety gives rise to IS) or they are underpinned by a common underlying cognitive and neurobiological mechanisms such as, for instance, impairments in different facets of self-regulation or imbalance between habit and goal-directed systems. These insights are particularly crucial given the impairing nature of both IS and anxiety in ASD, and recent longitudinal findings by Baribeau and colleagues [66] suggesting their stability or even increase during early and middle childhood in this population and findings suggesting that anxiety in ASD tends to be stable across the life-span [68].

## Limitations

The five datasets used here afforded a large as well as wide distribution of the RRB item scores. However, RBS-R scores used for establishing convergent and divergent validity of the derived factor scores were only available for a portion of the sample. Similarly, IQ and CBCL data were available only for a portion of participants. However, the sample size available to explore the pattern of relationships between derived RRB factor scores with other individual characteristics such as IQ, anxiety and externalizing problems was an order of magnitude larger than samples used in previous investigations. RMB SRS-2 factor contained only 2 items, and the majority of other derived factors had 3 items. Despite this limited item coverage, it has been shown that two, or even single-item indicators can be reliable [67, 69, 71] and this is further supported by the stability of the SRS-2 RMB factor (and other factors containing 3 items) across exploratory and confirmatory subsamples, as well as across sex and age subgroups. In addition, both the SCQ and the ADI-R were developed as screening and/or diagnostic instruments rather than for quantifying individual variability in the symptom severity and are therefore likely not sensitive enough to capture subtle symptom expressions observed below the diagnostic threshold, across a range of neurodevelopmental and neuropsychiatric disorders and in early normative development where distinct RMB and IS behaviors commonly occur (as discussed above). Therefore, further work is needed in order to appraise the viability of using the SCQ and ADI-R for this purpose. Ideally, quantitative comprehensive instruments optimized for capturing subtle variations in symptom expression and severity should be used to further our understanding of the phenomenological and mechanistic continuities and discontinuities in RRB across normative and atypical development, including ASD. Finally, it is possible that a small portion of participants overlapped across datasets, however, given the large sample size, overlapping cases are unlikely to have any impact on results.

## Conclusions

In summary, our findings demonstrate the capacity and the limitations of the SRS-2, SCQ and ADI-R to measure RRB in individuals with ASD. These measures were not specifically developed to capture distinct RRB domains; however, they are widely used in research and clinical practice and have been collected in several large datasets that are available to the research community. Therefore, the RRB factors derived here present an important resource for both re-analysis of the currently existing data and future investigations utilizing these measures. In particular, IS factor is relatively robust across all measures, encompassing three items for both the SRS-2 and the SCQ and five items for the ADI-R. The ADI-R RMB factor is also relatively robust; however, this factor is captured by only two SCQ and SRS-2 items. Finally, CI factor is only captured by the SRS-2, and the UI factor encompassing items such as fascination with sensory stimuli, fascination with parts of the object and interests unusual in terms of their content/subject emerged across the SCQ and the ADI-R, but not the SRS-2. CI and UI factors showed distinct correlations with factors such as age and IQ and therefore should be considered as separate in future investigations. Described factors showed good convergence with corresponding factors from a widely used dedicated RRB measure (RSB-R) and expected pattern of predicted external correlates (e.g., age, FSIQ, externalizing and internalizing symptoms). Therefore, RRB factors derived here can be used across both already collected data sets and newly planned studies to further our understanding of their relationship with a range of demographic, developmental and clinical correlates. Although two-item factors can be reliable, they nevertheless have a limited range which can somewhat limit their use in longitudinal and neurobiological research and although factors derived here can be used for such purposes, findings should be interpreted as preliminary, forming an initial first step towards future studies that would utilize detailed RRB instruments. Findings reported here provide important information for the development of future RRB questionnaire and interview measures. More specifically, our analysis demonstrated that the SCQ, SRS-2 and ADI-R provide a very limited sampling of sensory domains and very poor capture of the CI domain. This limitation is not specific to instruments analyzed here but also extends to dedicated RRB instruments such as the RBS-R and CY-BOCS-ASD. Although the RBQ-2 and the CRI-R have several sensory related items, they have not emerged as a separate factor in the factor analyses published thus far. Findings emphasizing the importance of distinguishing between interests that are unusual in terms of focus/flexibility and/or intensity rather than content and interests that are unusual in terms of content is particularly informative for the development of new instruments. Furthermore, the fact that sensory sensitivity factor emerged in the cross-measure ESEM that combined the SRS-2 and ADI-R items supports the viability of the sensory sensitivity as a separate domain linked to RRB. Additionally, new instruments should provide an in-depth sampling of each separate domain, with 10–15 items per subscale in order to provide an adequate range for different research and clinical purposes, in particular person-centered profiling and treatment tracking. New instruments will need to be designed and refined based on the gold standard measurement development standards (e.g., Patient-Reported Outcome Measurement Information System [PROMIS; 70]) and validated using the state-of-the-art psychometric approaches. Finally, our findings suggest that although ADOS is a valid and reliable diagnostic instrument, the coverage of RRB does not allow a fine-grained assessment of RRB domains. This finding emphasizes the need to develop new, dedicated observational assessment protocols specifically focused on RRB.

## Supplementary Information

**Additional file 1.** **Table S1**. Social Responsiveness Scale item loadings for exploratory models. **Table S2**. Social Communication Questionnaire item loadings for exploratory models. **Table S3**. Autism Diagnostic Interview-Revised item loadings for exploratory models. **Table S4**. Summary of Goodness of Fit Indices for Autism Diagnostic Interview-Revised Models based on “Ever” scores. **Table S5**. Cross-measure Exploratory Structural Equation Modelling factor loadings for four-factor solution. **Table S6**. Cross-measure Exploratory Structural Equation Modelling factor loadings for five-factor solution

## Data Availability

The datasets analyzed during the current study are available from the corresponding author on reasonable request.

## Abbreviations

ADI-R: Autism Diagnostic Interview-Revised
ADOS: Autism Diagnostic Observation Schedule
ASD: Autism Spectrum Disorder
CI: Circumscribed Interests
CRI: Childhood Routines Inventory
ESEM: Exploratory Structural Equation Modeling
FSIQ: Full-scale Intelligence Quotient
IS: Insistence on Sameness
RBS-R: Repetitive Behavior Scale-Revised
RMB: Repetitive Motor Behaviors
RRB: Restricted and Repetitive Behaviors
SCQ: Social Communication Questionnaire
SRS-2: Social Responsiveness Scale
UI: Unusual Interests
