# Supplementary material for: Using the big data approach to clarify the structure of restricted and repetitive behaviors across the most commonly used autism spectrum disorder measures
Source: Mol Autism. 2021 May 27;12:39. doi: 10.1186/s13229-021-00419-9 (PMC8162018; doi:10.1186/s13229-021-00419-9)
Supplement: Supplementary file 1 — Additional file 1. Table S1. Social Responsiveness Scale item loadings for exploratory models. Table S2. Social Communication Questionnaire item loadings for exploratory models. Table S3. Autism Diagnostic Interview-Revised item loadings for exploratory models. Table S4. Summary of Goodness of Fit Indices for Autism Diagnostic Interview-Revised Models based on “Ever” scores. Table S5. Cross-measure Exploratory Structural Equation Modelling factor loadings for four-factor solution. Table S6. Cross-measure Exploratory Structural Equation Modelling factor loadings for five-factor solution [file 13229_2021_419_MOESM1_ESM.docx]

**Supplement**

**Table S1. Social Responsiveness Scale item loadings for exploratory models**

| **Items** | **CI**  **Factor Loading (SE)** | **RMB**  **Factor Loading (SE)** | **IS**  **Factor Loading (SE)** |
| --- | --- | --- | --- |
| **4. Inflexible/rigid behaviors** | .13 (.01) | .26 (.01) | .45 (.01) |
| **20. Unusual sensory interests** | .02 (.001) | .75 (.01) | .07 (.01) |
| **24. Difficulties with changes in routine** | .09 (.009) | .15 (.01) | .63 (.01) |
| **28. Thinks about the same thing** | .86 (.02) | .02 (.006) | -.02 (.007) |
| **31. Fixated on certain topics/thoughts** | .58 (.02) | .01 (.009) | .27 (.02) |
| **39. Narrow range of interests** | .32 (.01) | .30 (.01) | .19 (.01) |
| **50. Motor mannerisms** | .04 (.009) | .67 (.01) | .00 (.01) |
| **61. Hard time changing one’s mind** | .11 (.01) | -.001 (.008) | .73 (.01) |

Note: CI= circumscribed interests; IS= insistence on sameness; RMB= repetitive motor behaviors; SE= standard error.

**Table S2. Social Communication Questionnaire item loadings for exploratory models**

| **Items** | **IS**  **Factor Loading (SE)** | **UI**  **Factor Loading (SE)** | **RMB**  **Factor Loading (SE)** |
| --- | --- | --- | --- |
| **7. Says the same thing over and over** | .68 (.05) | -.07 (.03) | .004 (.03) |
| **8. Rituals** | .59 (.05) | .15 (.04) | .12 (.03) |
| **11. Unusual interests (e.g. traffic lights)** | .18 (.04) | .73 (.05) | .00 (.02) |
| **12. Interest in parts of a toy/object** | -.03 (.03) | .58 (.06) | .33 (.05) |
| **13. Overly intense interests in otherwise typical topics and/or objects** | .44 (.04) | .28 (.04) | -.006 (.03) |
| **14. Unusual sensory interests** | .12 (.03) | .38 (.04) | .27 (.04) |
| **15. Motor mannerisms** | -.02 (.03) | .15 (.06) | .60 (.06) |
| **16. Complex body movements** | .08 (.02) | -.02 (.03) | .80 (.06) |
| **18. Attachment to toys and/or objects** | .15 (.03) | .31 (.04) | .13 (.04) |

Note: IS= insistence on sameness; RMB= repetitive motor behaviors; SE= standard error; UI= unusual interests.

**Table S3. Autism Diagnostic Interview-Revised item loadings for exploratory models**

| **Items** | **UI**  **Factor Loading (SE)** | **IS**  **Factor Loading (SE)** | **RMB**  **Factor Loading (SE)** |
| --- | --- | --- | --- |
| **39. Verbal rituals** | .07 (.04) | .51 (.02) | -.02 (.04) |
| **67. Unusual preoccupations** | .24 (.04) | .11 (.03) | .16 (.04) |
| **68. Circumscribed interests** | -.06 (.03) | .47 (.02) | -.11 (.04) |
| **69. Repetitive object use/interest in parts of objects** | .73 (.05) | -.04 (.02) | .10 (.04) |
| **70. Compulsions/rituals** | .15 (.03) | .49 (.02) | .13 (.04) |
| **71. Unusual sensory interests** | .53 (.05) | -.03 (.02) | .25 (.04) |
| **74. Difficulties with changes in own routines and/or environment** | .03 (.02) | .69 (.02) | .05 (.03) |
| **75. Resistance to trivial changes in the environment not related to the individual** | .09 (.04) | .56 (.03) | .07 (.04) |
| **76. Unusual attachment to objects** | .34 (.05) | .24 (.03) | -.04 (.05) |
| **77. Hand and finger mannerisms** | .07 (.04) | -.05 (.02) | .65 (.06) |
| **78. Complex mannerisms** | .20 (.05) | .01 (.02) | .45 (.05) |
| **79. Midline hand movements** | -.01 (.06) | .15 (.04) | .37 (.06) |

Note: IS= insistence on sameness; RMB= repetitive motor behaviors; SE= standard error; UI= unusual interests.

**Table S4. Summary of Goodness of Fit Indices for Autism Diagnostic Interview-Revised Models based on “Ever” scores**

| **Model** |  | **χ^2^** | **CFI** | **TLI** | **RMSEA** | **SRMR** |
| --- | --- | --- | --- | --- | --- | --- |
| **Ever Scores** |  |  |  |  |  |  |
| EFA Unidimensional | Sample 1 | 1783.42** | .694 | .627 | .087** | .081 |
| EFA 2-Factor | Sample 1 | 236.119** | .966 | .948 | .032 | .030 |
| EFA 3-Factor | Sample 1 | 128.919** | .983 | .966 | .026 | .021 |
| ESEM 3-Factor | Sample 2 | 95.138** | .988 | .973 | .026 | .020 |

Note: *p< .01; **p< .001; ^a^Age Group 1: 2-12 years; ^b^Age Group 2: 13 years and above; CFA: Confirmatory Factor; CFI: Comparative Fit Index; ESEM: Exploratory Structural Equation Modelling; RMSEA= Root Mean Square Error of Approximation; SRMR= Standardized Root Mean Square Residual; TLI= Tucker-Lewis Index.

**Table S5. Cross-measure Exploratory Structural Equation Modelling factor loadings for four-factor solution**

| **Items** | **CI** | **RMB** | **UI** | **IS + SS** |
| --- | --- | --- | --- | --- |
| **SRS 28. Thinks or talks about the same thing over and over** | **0.823** | 0.077 | -0.042 | -0.035 |
| **SRS 31. Can’t get mind of something** | **0.865** | 0.056 | 0.038 | 0.034 |
| **SRS 42. Sensitive to sounds** | 0.087 | 0.298 | 0.006 | **0.62** |
| **SRS 50. Repetitive motor behavior** | 0.103 | **0.876** | 0.054 | 0.024 |
| **SRS 61. Inflexible** | **0.514** | -0.019 | 0.12 | 0.203 |
| **ADI-R 67. Unusual preoccupations** | 0.071 | -0.001 | **0.378** | 0.041 |
| **ADI-R 68. Circumscribed interests** | **0.392** | -0.047 | -0.062 | 0.269 |
| **ADI-R 69. Repetitive and/or interest in objects** | -0.002 | 0.129 | **0.76** | -0.071 |
| **ADI-R 70. Compulsions** | 0.213 | -0.018 | 0.263 | **0.308** |
| **ADI-R 71. Unusual sensory interests** | -0.083 | 0.184 | **0.605** | 0.061 |
| **ADI-R 72. Sensitivity to noise** | -0.047 | 0.174 | -0.028 | **0.616** |
| **ADI-R 73. Abnormal response to sensory stimuli** | -0.014 | -0.023 | 0.183 | **0.5** |
| **ADI-R 74. Difficulties with changes in own routines and/or environment** | 0.273 | -0.097 | 0.122 | **0.503** |
| **ADI-R 75. Resistance to trivial changes in the environment not related to the individual** | 0.118 | -0.127 | 0.183 | **0.489** |
| **ADI-R 76. Unusual attachment to objects** | 0.143 | 0.004 | 0.269 | 0.091 |
| **ADI-R 77. Hand mannerisms** | -0.062 | **0.653** | 0.146 | 0.003 |
| **ADI-R 78. Complex mannerisms** | 0.017 | **0.402** | 0.267 | 0.039 |
| **ADI-R 79. Midline hand movements** | 0.097 | 0.156 | 0.164 | 0.152 |

Note: CI= circumscribed interests; IS= insistence on sameness; RMB= repetitive motor behaviors; SS= sensory sensitivities; UI= unusual interests.

**Table S6. Cross-measure Exploratory Structural Equation Modelling factor loadings for five-factor solution**

| **Items** | **CI** | **RMB** | **SS** | **UI** | **IS** |
| --- | --- | --- | --- | --- | --- |
| **SRS 28. Thinks or talks about the same thing over and over** | **0.765** | 0.061 | 0.02 | -0.042 | 0.055 |
| **SRS 31. Can’t get mind of something** | **0.878** | 0.002 | 0.1 | 0.057 | 0.024 |
| **SRS 42. Sensitive to sounds** | 0.116 | 0.144 | **0.743** | 0.025 | 0.009 |
| **SRS 50. Repetitive motor behavior** | 0.075 | **0.895** | 0.095 | 0.022 | -0.013 |
| **SRS 61. Inflexible** | **0.442** | 0.014 | 0.082 | 0.061 | 0.279 |
| **ADI-R 67. Unusual preoccupations** | 0.064 | -0.001 | 0.013 | **0.359** | 0.104 |
| **ADI-R 68. Circumscribed interests** | **0.324** | -0.032 | 0.137 | -0.101 | 0.254 |
| **ADI-R 69. Repetitive and/or interest in objects** | 0.036 | 0.109 | 0.009 | **0.733** | 0.002 |
| **ADI-R 70. Compulsions** | 0.106 | 0.059 | 0.051 | 0.172 | **0.444** |
| **ADI-R 71. Unusual sensory interests** | -0.031 | 0.113 | 0.152 | **0.636** | -0.041 |
| **ADI-R 72. Sensitivity to noise** | -0.017 | 0.026 | **0.632** | 0.012 | 0.062 |
| **ADI-R 73. Abnormal response to sensory stimuli** | -0.024 | -0.088 | **0.406** | 0.174 | 0.212 |
| **ADI-R 74. Difficulties with changes in own routines and/or environment** | 0.062 | 0.035 | 0.091 | -0.06 | **0.769** |
| **ADI-R 75. Resistance to trivial changes in the environment not related to the individual** | -0.004 | -0.051 | 0.174 | 0.078 | **0.516** |
| **ADI-R 76. Unusual attachment to objects** | 0.107 | 0.032 | 0.01 | 0.227 | 0.177 |
| **ADI-R 77. Hand mannerisms** | -0.089 | **0.65** | 0.023 | 0.134 | 0.025 |
| **ADI-R 78. Complex mannerisms** | -0.009 | **0.409** | 0.03 | 0.241 | 0.084 |
| **ADI-R 79. Midline hand movements** | 0.056 | 0.18 | 0.061 | 0.124 | 0.179 |

Note: CI= circumscribed interests; IS= insistence on sameness; RMB= repetitive motor behaviors; SS= sensory sensitivities; UI= unusual interests.
